# Neonatal resuscitation guideline adherence: simulation study and framework for improvement

**DOI:** 10.1007/s00431-020-03693-6

**Published:** 2020-05-29

**Authors:** Mathijs Binkhorst, Irene van de Wiel, Jos M. T. Draaisma, Arno F. J. van Heijst, Tim Antonius, Marije Hogeveen

**Affiliations:** 1 Department of Neonatology, Amalia Children’s Hospital, Radboud Institute for Health Sciences (RIHS), Radboud University Medical Center, P.O. Box 9101, 6500 HB Nijmegen, the Netherlands; 2 Radboudumc Health Academy, Radboud University Medical Center, Nijmegen, the Netherlands; 3 Department of Pediatrics, Amalia Children’s Hospital, Radboud University Medical Center, Nijmegen, the Netherlands

**Keywords:** Cardiopulmonary resuscitation, Newborn, Pediatrician, Simulation training, Guideline adherence

## Abstract

We wanted to assess newborn life support (NLS) knowledge and guideline adherence, and provide strategies to improve (neonatal) resuscitation guideline adherence. Pediatricians completed 17 multiple-choice questions (MCQ). They performed a simulated NLS scenario, using a high-fidelity manikin. The literature was systematically searched for publications regarding guideline adherence. Forty-six pediatricians participated: 45 completed the MCQ, 34 performed the scenario. Seventy-one percent (median, IQR 56–82) of the MCQ were answered correctly. Fifty-six percent performed inflation breaths ≤ 60 s, 24% delivered inflation breaths of 2–3 s, and 85% used adequate inspiratory pressures. Airway patency was ensured 83% (IQR 76–92) of the time. Median events/min, compression rate, and percentage of effective compressions were 138/min (IQR 130–145), 120/min (IQR 114–120), and 38% (IQR 24–48), respectively. Other adherence percentages were temperature management 50%, auscultation of initial heart rate 100%, pulse oximeter use 94%, oxygen increase 74%, and correct epinephrine dose 82%. Ten publications were identified and used for our framework. The framework may inspire clinicians, educators, researchers, and guideline developers in their attempt to improve resuscitation guideline adherence. It contains many feasible strategies to enhance professionals’ knowledge, skills, self-efficacy, and team performance, as well as recommendations regarding equipment, environment, and guideline development/dissemination.

*Conclusion*: NLS guideline adherence among pediatricians needs improvement. Our framework is meant to promote resuscitation guideline adherence.

**Table:** 

**What is Known:** • *Inadequate newborn life support (NLS) may contribute to (long-term) pulmonary and cerebral damage.* • *Video-based assessment of neonatal resuscitations has shown that deviations from the NLS guideline occur frequently; this assessment method has its audiovisual shortcomings.*	
**What is New:** • *The resuscitation quality metrics provided by our high-fidelity manikin suggest that the adherence of Dutch general pediatricians to the NLS guideline is suboptimal.* • *We constructed a comprehensive framework, containing multiple strategies to improve (neonatal) resuscitation guideline adherence.*

**Electronic supplementary material:**

The online version of this article (10.1007/s00431-020-03693-6) contains supplementary material, which is available to authorized users.

## Introduction

Approximately 5–10% of all newborns require support of transition to initiate breathing and aerate the lungs; true resuscitation at birth, including chest compressions and medications, occurs significantly less often [1–7]. Inadequate initial cardiorespiratory support may inflict pulmonary damage and may conduce to ongoing hypoxia/ischemia, possibly aggravating cerebral injury. The European Resuscitation Council (ERC) and American Heart Association (AHA) publish guidelines for the resuscitation of newborns at birth [1, 8, 9]. Healthcare professionals qualified to perform neonatal resuscitations are expected to abide by these guidelines. By attending the Newborn Life Support (NLS) and/or European Pediatric Advanced Life Support (EPALS) courses of the ERC, or the Neonatal Resuscitation Program (NRP) outside Europe, professionals can acquire and maintain the knowledge and skills required for guideline adherence. In the Netherlands, the NLS course is obligatory for pediatric residents. Many Dutch pediatricians also participate in this course. There is some, though limited evidence that structured neonatal resuscitation training improves patient outcomes [3, 4, 10].

A prerequisite for the positive effect of resuscitation guidelines on patient outcomes is adequate adherence to these guidelines. Previous studies looked at the adherence to neonatal resuscitation guidelines using video recording of delivery room management. Carbine et al*.* reported that 54% of neonatal resuscitations deviated from guideline recommendations [11]. Other studies also revealed insufficient compliance with the guidelines [2, 3, 6, 10, 12]. More complex resuscitations were associated with poorer adherence [2, 11]. Commonly reported deviations were overly vigorous stimulation, undue/inadequate suctioning, inadequate/unnecessary positive pressure ventilation, unjustified delivery of oxygen, prolonged/multiple intubation attempts, not completing tasks within the allocated time intervals, and overestimated Apgar scores. Most studies could not reliably report on the adequacy of chest compressions (CC), because resuscitations requiring compressions were infrequent. Crew resource management (CRM) skills during neonatal resuscitations also deserved attention [6, 10].

Video-based assessment of simulated or real-life neonatal resuscitations proved to be feasible and informative in the aforementioned studies. However, video recording, as the only means of assessment, has its audiovisual limitations [13], and does not provide objective, quantitative data on vital physiological parameters [14]. Therefore, we evaluated the adherence of Dutch pediatricians to the NLS guideline of the ERC in a simulated setting, using a scenario running on a model-driven, high-fidelity neonatal patient simulator. This allowed for the measurement of several resuscitation quality metrics, including airway patency, applied airway pressures, and CC. We also assessed pediatricians’ NLS knowledge and reviewed strategies to improve (neonatal) resuscitation guideline adherence.

## Materials and methods

Data were collected during a 3-day continuing medical education (CME) event for general pediatricians at our center. The main theme of the event did not involve (neonatal) resuscitation, so participants did not specifically prepare for participation in our study. This study took place just prior to the latest update of the ERC guidelines in 2015. Hence, we used the 2010 ERC guideline (and its Dutch equivalent) as a reference [1]. The only changes in the 2015 ERC guideline relevant to this study were (1) the more explicit recommendation to apply a higher peak inspiratory pressure (PIP) (i.e., 30 cm H_2_O) during inflation breaths in term newborns, (2) the gestational age below which plastic wrapping is indicated (< 32 weeks instead of < 28 weeks), and (3) the option to start with 30% oxygen in preterm infants [8].

### Background characteristics

We gathered information on the pediatricians’ sex, working experience, hospital type and location, NLS course attendance, and time since last NLS certification. Note that pediatricians are fully trained consultants in the Netherlands.

### Knowledge assessment

On each day, a subset of pediatricians attending the CME event completed a knowledge test, all at the same time. This test consisted of 23 NLS-related multiple-choice questions (MCQ), based on the 2010 ERC guideline. The test was developed by MH and critically appraised by TA. Both are qualified and experienced medical educators. Pediatricians were not acquainted with their knowledge test results prior to participation in the simulation scenario in order to study their NLS skills without specific preparation.

### Simulation scenario

Following the knowledge test, (the same subset of) pediatricians participated in a NLS scenario in the simulation facility of our level III perinatal care center. The scenario was not scripted. Participants were requested to perform a real-time resuscitation at birth according to the guidelines, so the NLS algorithm itself served as “script.” Beforehand, the simulation room and available equipment were demonstrated. Participants received a detailed orientation to the neonatal patient simulator, including an explanation regarding the real-time preparation, insertion, and use of an umbilical venous catheter (UVC). Each scenario started with a briefing: a term newborn with an appropriate weight for gestational age, born after an uneventful pregnancy, with clear amniotic fluid, required resuscitation at birth. Standard delivery room necessities, including warm towels and a hat, were provided. The radiant warmer was already switched on. Vitals were shown on a standard patient monitor. A non-obstructive nurse was present from the start of the scenario. This nurse acted as in real clinical care, but only on the participant’s request; the nurse did not prompt or initiate any actions independently. Participants could summon one or more colleagues for back-up assistance. The scenario ended when the patient recovered. Recovery was defined as a heart rate > 100/min, which occurred once the simulator had detected an adequate minute ventilation and after epinephrine had been administered. To avoid a learning curve, there was no collective debriefing and no possibility to watch each other’s performance. Pediatricians did receive individual feedback. The participants were asked not to discuss the contents of the scenario (and knowledge test) with the remaining CME event participants.

All simulations were supervised by MH and/or TA, sitting in a control room separated from the simulation room by a one-way mirror. Both are neonatologists and NLS instructors with extensive experience in high-fidelity video-assisted real-time simulation training. They provided brief answers to queries of the participants via the intercom; there were no time-consuming discussions influencing time responses. No extra cues or suggestions were given. The simulations were videotaped with multiple ceiling-mounted cameras for later assessment.

An originally low-fidelity manikin (Newborn Anne, Laerdal Benelux, the Netherlands) was customized and provided with various recording features by TA (an expert in simulation models), transforming it into a model-driven, high-fidelity neonatal patient simulator. Tidal volumes, airway pressures, and CC characteristics were calibrated using flow and pressure sensors. This simulator was pre-programmed in such a way that physiological responses to resuscitative interventions occurred automatically, without instructor interference, resulting in standardized effects of resuscitative efforts among the simulations [15]. The simulator provided the following resuscitation quality metrics: start and duration of inflation breaths; PIP; duration of airway patency; start, rate, and effectiveness of CC; events/min; time to epinephrine administration, and time to recovery. The simulator determined CC rate by measuring the time interval between CC instead of counting the number of CC/min. The corresponding recommended CC rate is thus 100–120/min instead of 90/min (i.e., 120 events/min minus 30 ventilations/min).

All videotaped scenarios were assessed by IvdW to evaluate adherence to readily observable items of the NLS algorithm. A checklist was used to assess whether the following tasks were done in the right way and sequence: drying the newborn, removal of wet towels, hat placement, auscultation of initial heart rate, pulse oximetry use, increase in oxygen when starting CC, and epinephrine administration.

### Literature search

Several strategies to ameliorate (neonatal) resuscitation guideline adherence could be extracted from the publications that were already identified as references for our manuscript [2, 4, 7, 10–23]. We endeavoured to construct a comprehensive framework with recommendations to improve guideline adherence for cardiopulmonary resuscitation (CPR) in general, and for NLS in particular. Therefore, a systematic literature search was performed to identify additional publications. The following search strategy was used in PubMed:

1. Reviews on improvement of guideline adherence in general: (“Guideline Adherence” [Mesh]) AND ((((implementation) OR enhance) OR increase) OR improv*) (* denotes truncation symbol). Limits: English, humans, review.
2. Publications on improvement of resuscitation guideline adherence: (((“Guideline Adherence” [Mesh]) AND ((((implementation) OR enhance) OR increase) OR improv*))) AND ((("Cardiopulmonary Resuscitation" [Mesh]) OR "Resuscitation" [Mesh]) OR life support). Limits: English, humans, review, systematic reviews, meta-analysis, randomized controlled trial.
3. “Similar articles” of the publication by Cabana et al*.* [16]. Limits: English, humans, reviews.

MB and MH independently screened the titles of candidate articles. If titles were unclear, abstracts and/or full texts were appraised for eligibility. The reference lists of relevant articles were also reviewed. Differences in the selection of articles were discussed between MB and MH; together, they decided on the final selection of the articles in question.

### Statistical analysis

Resuscitation quality metrics were not normally distributed and therefore presented as median with interquartile range (IQR) and range. Descriptive statistics were used for the video observations and the MCQ. Multiple linear regression analysis with backward selection was performed, with working years in pediatrics, years elapsed since last NLS course, and percentage of correct MCQ answers as independent variables, and inflation time, percentage of effective compressions, percentage of airway patency, CC rate, and events/min as outcome parameters, respectively. Statistical analyses were performed with SPSS for Windows version 22.0 (IBM, Armonk, NY, USA).

Study participation was voluntary and written informed consent was obtained from all participants. The Institutional Review Board concluded that study approval was not necessary, since human subjects were not exposed to medical activities (file number 2018-4428).

## Results

### Background characteristics

Forty-six pediatricians participated. Due to time constraints and the fact that pediatricians, who provided back-up assistance for a colleague, did not start their own scenario anymore, only 34 participated in the scenario. Forty-five pediatricians completed the MCQ. Participants worked in 24 different Dutch hospitals (23 general, 1 academic). Twenty-three participants (50%) were male. Median years of working experience in pediatrics was 13 (IQR 8–21). Twenty-one (46%) and 39 (85%) participants had attended the NLS and Advanced Paediatric Life Support (APLS) course, respectively. A median of 4 years (IQR 2–5) and 5 years (IQR 2–7) had elapsed since last NLS and APLS certification, respectively. There were no significant differences between “NLS providers” and “APLS providers” regarding the knowledge test results and scenario performances mentioned. Dutch general pediatricians are exposed to approximately 1–2 real neonatal resuscitations each year.

### Knowledge assessment

MCQ results are presented in Table 1. A version of this table with all answer options to the MCQ can be found in Online Resource 1. Six questions were excluded from analysis, since they did not strictly pertain to information contained in the NLS course manual or guideline. Participants answered a median of 71% (IQR 56–82) of the questions correctly. Questions regarding laryngeal mask airway use, delayed cord clamping, ventilation rate, compression/ventilation ratio, and events/min were answered correctly by ≤ 40% of the pediatricians.

**Table 1:** Knowledge test results (*n*=45)

No.	Question	Correct
1	Below which gestational age is plastic wrapping recommended?	84.4%
2	What are the possible consequences of hypothermia directly after birth?	80.0%
3	Below which heart rate is it unreliable to feel cord pulsations?	57.8%
4	Is colour assessment essential and reliable to judge oxygenation?	80.0%
5	What are the correct head position and airway opening manoeuvres for a newborn?	95.6%
6	How to determine the correct size of an oropharyngeal airway?	77.8%
7	Above which gestational age/weight can a LMA be considered?	33.3%
8	How should the initial inflation breaths be performed?	82.2%
9	What is the correct rate of ventilations in the absence of spontaneous breathing?	35.6%
10	What is an acceptable pre-ductal oxygen saturation at 5 min?	66.7%
11	How/at which site should a pulse oximeter be applied?	66.7%
12	What are the correct compression/ventilation ratio and number of events per minute?	40.0%
13	Below which heart rate should chest compressions be started?	82.2%
14	When should the FiO_2_ be increased, if not already done before? ^a^	75.6%
15	What is the correct dose of epinephrine?	68.9%
16	What is the recommended administration route of epinephrine?	95.6%
17	In which babies is delayed cord clamping (1 min) recommended?	40.0%

*FiO*_2_, fraction of inspired oxygen; *LMA*, laryngeal mask airway

^a^Although not evidence-based, the administration of supplementary oxygen at the start of chest compressions is considered to be ‘sensible’ according to the ERC guideline and it is an actual prescription in the Dutch NLS guideline

### Simulation scenario

Resuscitation quality metrics are shown in Table 2. None of the participants performed < 100 CC/min; 17 (50%) delivered > 120 CC/min. The median time interval between initiation of CC and epinephrine administration was 255 s (IQR 206–348). Scores for the other items, as obtained by video observation, are presented in Table 3. In both tables, we provided the error type associated with each item to make a clear distinction between inadequate skill performance (errors of commission) and inadequate execution of the consecutive steps of the algorithm (errors of omission). Multiple linear regression analysis did not produce clinically relevant results.

**Table 2:** Resuscitation quality data as provided by the neonatal patient simulator (*n* = 34)

	Median (IQR)	Range	ERC guideline	Correct *n* (%)	Associated error type ^a^
Start inflation breaths (sec)	55 (47-72)	36-206	≤ 60	19 (56%)	Commission
Inflation breath duration (sec)	1.67 (1.47-1.67)	1.08-2.83	2-3	8 (24%)	Commission
Maximum PIP (cm H_2_O)	19 (18-19)	15-37	20 ^b^	29 (85%)	Commission
Airway open (% of time)	83 (76-92)	39-100	100 ^c^	3 (8.8%)	Commission
Start CC (sec)	108 (90-151)	67-254	-	-	-
CC (per min)	120 (114-120)	102-142	100-120 ^d^	17 (50%)	Commission
Effective CC (%)	38 (24-48)	10-69	100 ^c^	0 (0%)	Commission
Events per minute	138 (130-145)	124-172	120	4 (11.8%)	Commission
Administration of epinephrine (sec)	377 (320-497)	211-677	-	-	-
Time to recovery (sec)	444 (388-565)	271-719	-	-	-

*CC*, chest compressions; *ERC*, European Resuscitation Council; *IQR*, interquartile range, *PIP*, peak inspiratory pressure

^a^According to Yamada et al. [2]. Errors of commission are interventions that are not indicated, not timely done, or not adequately performed. Errors of omission are interventions that are indicated, but not performed

^b^The 2010 ERC guideline literally stated that ‘an initial inflation pressure of 20 cm H_2_O may be effective, but 30-40 cm H_2_O or higher may be required in some term babies [1].’ The 2010 Dutch guideline on newborn life support more strictly prescribed an initial PIP of 20 cm H_2_O

^c^Not literally mentioned in the ERC guideline, but evidently the desired percentage

^d^Although the effective number of compressions per min should be 90, due to intervening ventilations, the recommended rate is 100-120 CC/min

**Table 3:** Scores for items assessed by video observation (*n*=34)

Item	Done, *n* (%)	Associated error type ^a^
Drying the newborn	32 (94%)	Omission
Removal of wet towels	18 (53%)	Omission
Hat placement	23 (68%)	Omission
Temperature management ^b^	17 (50%)	Omission
Initial heart rate assessment ^c^	34 (100%)	Omission
Correct application of pulse oximeter	32 (94%)	Omission
Increase in oxygen concentration ^d^	25 (74%)	Omission
Correct epinephrine dose ^e^	28 (82%)	Commission

^a^According to Yamada et al. [2]. Errors of commission are interventions that are not indicated, not timely done, or not adequately performed. Errors of omission are interventions that are indicated, but not performed

^b^All 3 items (drying, towels, and hat) combined

^c^Auscultation was required for heart rate assessment; palpation of umbilical pulse was disapproved in the presence of bradycardia

^d^At the start of chest compressions. Although not evidence-based, increasing the oxygen concentration at the initiation of chest compressions is considered to be ‘sensible’ according to the ERC guideline and it is an actual instruction in the Dutch NLS guideline

^e^10 micrograms/kg intravenously (recommended route) or 50-100 micrograms/kg endotracheally (not recommended, only as a last resort)

### Literature search

Our search strategy yielded the following results:

1. 925 hits, four studies selected: 2 systematic meta-reviews [24, 25], 1 systematic review [26], 1 qualitative focus group study [27].
2. 190 hits, four studies selected: 3 randomized controlled trials [28–30], 1 retrospective cohort study [31].
3. 43 hits, two studies selected: both systematic reviews [32, 33].

From these ten studies, we extracted additional strategies to improve (resuscitation) guideline adherence. The abridged versions of all these strategies were transferred to our framework (Table 4). The main categories of the framework (characteristics of the professionals, environment/equipment, and guidelines) were adapted from Francke et al. [24].

**Table 4:** Framework for improvement of (neonatal) resuscitation guideline adherence [2, 4, 7, 10–33]

Head		
Characteristics of the professionals	Adequate acquisition of knowledge and skills	Examples / extra information
	Improve factors influencing resuscitation course participation	Time constraints, costs, distance, enough courses
	Guarantee that all resuscitation team members are appropriately certified	Compulsory NLS certification for all personnel involved in neonatal resuscitation, incl. residents
	Organize local or regional *in situ* simulation training sessions	Outreach program
	Rehearse individual technical skills with hands-on practice	Focused practice using skill stations
	Familiarize all resuscitation team members with the equipment	Especially with new and complex devices
	Combine relevant aspects of ‘deliberate practice’ and ‘mastery learning’	See references [15] and [17]
	Adequate retention of knowledge and skills	
	Ensure regular clinical exposure to resuscitations	By adapting shifts and rotations
	Refresher course participation	At least every 6-12 months
	Attend bedside booster sessions	At least every 3 months
	Regular engagement in mental rehearsal (‘imagined practice’)	Visualization of NLS performance
	Make a team member responsible for ‘staying up-to-date’	Membership of a resuscitation council
	Organize local or regional educational meetings to increase awareness of and familiarity with (updates of) the guidelines	CME events, journals clubs, video conferences, esp. for senior generalists in small centers
	Apply the principle of ‘spaced learning’ with increasing difficulty	See reference [15]
	Feedback on performance after resuscitations	
	Formative assessment with error-specific feedback	By experienced instructors with feedback skills
	Briefing and (facilitated) debriefing	Before and after all real and simulated scenarios
	Organize video review sessions	Video recordings of delivery room management
	Team performance	
	Provide training in CRM skills Standardized communication techniques	Communication of heart rate to lead resuscitator
	Leadership	To delegate tasks to decrease individual workload
	Team work Situational awareness	To identify roles and responsibilities
	Appoint a task-free observer to oversee the resuscitation scene	In control of the (electronic) decision support tool
	Ensure an adequate composition of the resuscitation team	Skilled team members may decrease the workload of the lead resuscitator
	Self-efficacy	
	Use methods to increase the self-efficacy of resuscitation team members To enhance access to knowledge and skills in spite of stress and challenges To increase the likelihood of initiating and persisting in resuscitative tasks To improve the transfer of skills learned during training to clinical practice	Methods: personal performance mastery experiences, verbal persuasion, observational learning (‘perfect demonstrations’), help with controlling emotions (see reference [22])
Characteristics of the environment/equipment	Equipment: prompts and aids to decrease cognitive load	Examples / extra information
	Equipment and performance checklists Posters displaying relevant algorithms Pocket cards containing relevant algorithms Relevant algorithms on smart phones and tablets	Should be available on site
	Metronomes	For the correct compression rate
	Timers indicating specific time intervals	A beep every 30 sec during compressions
	Electronic decision support tools with audiovisual prompts	See reference [18]
	Augmented/mixed reality devices	Hololens, Google Glass (see reference [30])
	Early activated, synchronous audio-video telemedicine consultation of a remote expert	Teleneonatology, esp. for preterm deliveries in community hospitals (see references [29] and [31])
	Equipment: real-time quantitative feedback devices	
	ECG, pulse oximeter, temperature probe Respiratory function monitor	PIP, PEEP, Vt, FiO_2_, EtCO_2_, mask/tube leak, airway patency, spontaneous breathing activity
	Q-CPR (development of accelerometers suitable for newborns) All feedback parameters ideally integrated and displayed on one screen	CC rate, depth, recoil, position of thumbs
	Environment	
	Ensure an appropriate resuscitation environment Ensure sufficient personnel resources	Adequate ambient temperature, enough space
	Resolve organizational constraints Endeavour guideline agreement among colleagues	Provision of essential devices, resources, facilities
	Discuss factors influencing guideline adherence with colleagues	Personal autonomy, individual experience, attitudes, and beliefs
Characteristics of the guidelines	Guideline development and content	Examples / extra information
	Increase the quality of evidence supporting guideline recommendations Assemble evidence showing that adherence improves patient outcomes	A clear scientific base promotes adherence
	Ensure that guideline recommendations are feasible	First 60 sec of NLS algorithm is a challenge
	Create simple, concise, and convenient guidelines, avoid complexity	Less text, more figures/algorithms,no ambiguities
	Use mnemonics to facilitate recollection	MRSOPA
	Ensure that local, regional, national, and international guidelines are aligned	ABC versus CAB sequence
	Provide guidance for tailored interventions	For comorbidities and specific circumstances (e.g. CDH, extreme prematurity, fetal hydrops)
	Compose guideline writing group of credible, representative experts and opinion leaders, but also of end users from different disciplines	Nurses, residents, general pediatricians
	Use instruments to assess guideline quality	Most notably, the AGREE II instrument
	Guideline dissemination and implementation	
	Use active, multi-faceted implementation strategies	Educational outreach, interactive education
	Avoid passive, traditional dissemination strategies	Websites, conferences, didactic lectures, emails

## Discussion

This study revealed that the adherence of Dutch pediatricians to the NLS guideline was suboptimal. In line with the publication by Yamada et al*.* [2], guideline deviations can be separated into errors of commission and errors of omission. We mainly encountered errors of commission as far as technical skills (compressions and ventilations) were concerned. Inflation breaths were started too late, they lasted too short, continuous airway patency was infrequently achieved, and the majority of CC were ineffective. On the other hand, inspiratory pressures and epinephrine dose were mostly correct. Considering the fact that CC rate and events/min were comparatively high, it appears that rapidness occurred at the expense of effectiveness. Errors of omission occurred less often. A quarter of the pediatricians did not increase the amount of oxygen when CC were initiated, as prescribed in the Dutch NLS guideline and suggested in the ERC guideline. Only half of them took full measures to maintain body temperature. Pulse oximetry was applied by the majority of our participants and all assessed initial heart rate by auscultation. Looking at the error types, healthcare professionals are apparently in need of reminders/prompts to ensure execution of all steps of the algorithm, whereas they require real-time quantitative feedback to guide their performance of technical skills.

Overall, it was interesting to witness a large variation in algorithm execution and skill performance among the pediatricians. For example, time to recovery varied between 4.5 and 12 min. Our findings substantiate the results of previous researchers in that neonatal resuscitation guideline adherence is rather low, and that ventilation errors occur frequently. In addition, we found that circulatory support also needs improvement. The ventilatory flaws are perhaps more intriguing than the circulation-related inadequacies. After all, pediatricians are more exposed/used to assisted ventilation than to compressions and medications, as (respiratory) support of transition is needed far more often than extensive resuscitation.

NLS knowledge was also suboptimal. Seventy-one percent of the multiple-choice questions were answered correctly. The cut-off point for passing the knowledge assessment of the NLS course is 80%. From a database, held by the Dutch Resuscitation Council and the Dutch Foundation for the Emergency Medical Care of Children, containing data from 2016 to 2019, we learned that 97% of all Dutch NLS participants passed the post-course MCQ. Median (IQR) test scores on the pre-course and post-course MCQ were 90% (86–96%) and 90% (86–94%), respectively. These percentages are higher than the 71% found in our study, probably because the pre-course and post-course results were achieved following manual reading and the NLS course, respectively, whereas pediatricians were tested ad hoc in the current study. Pediatric residents at the University of Colorado (USA) had a pass rate of 79 ± 3% on a pretest that assessed NRP knowledge [17]. All these residents reviewed NRP in the preceding 6 months, so they had a relatively recent booster. Some questions in our MCQ, e.g., the ones on laryngeal mask airway use and delayed cord clamping, were perhaps a little less essential for the practice of NLS by general pediatricians at that time. However, various pediatricians appeared to be unaware of important information regarding ventilations and compressions.

The NLS certification rate of our participants was comparatively low (46%). The majority (85%) of our participants did attend the APLS course. However, the Dutch APLS course only contains background information on and a demonstration of NLS, but does not incorporate practical rehearsal of NLS skills. Moreover, a median of 4–5 years had elapsed since our participants attended their last certified NLS or APLS course. The recertification interval for NLS and APLS is 4 years in the Netherlands. Unfortunately, we were not informed about the rate and nature of the neonatal resuscitation simulation training that our participants attended at their local hospitals. We know that they usually rehearse NLS scenarios at least once a year using a lower fidelity manikin and environment. In view of our results, pediatricians probably need to follow more frequent booster training to maintain their resuscitation capabilities. Annual refreshers may not be sufficient, for it is well known that resuscitation skills deteriorate within 3–6 months after initial training without regular practice [34].

The identification of guideline deviations is especially important in planning interventions to optimize algorithm adherence and quality of skill performance [2, 3]. Instead of only focusing on errors, we intended to come up with solutions as well. We therefore decided to create the framework as depicted in Table 4. This framework is meant to inspire clinicians, researchers, medical technicians, manufacturers, medical educators, policy makers, and course and guideline developers. It is not a formally validated tool to be implemented as a whole at any particular department. It should serve as a guide or “checklist” for institutions and departments that seek to enhance (neonatal) resuscitation guideline adherence. Departments may select the interventions that are relevant and feasible for them. The framework contains recommendations to improve both the stepwise execution of the algorithm and the performance of the skills therein, in order to redeem errors of omission and commission, respectively. For example, pocket cards, prompts, mnemonics, and decision support tools may ameliorate the stepwise process, while regular hands-on practice with feedback (devices) and the provision of correct equipment are measures to ensure adequate skill performance. Having incorporated a column with examples and/or extra information, we believe that our framework is self-explanatory. Some of the strategies are quite innovative and require advanced technology to effectuate them. However, many other strategies are less resource-demanding and can be realized in basically every hospital. Strategies that involve the use of video recording in the delivery room may raise ethical concerns [14]. These should be addressed before implementation. In spite of the framework’s size, we would like to emphasize that one should not lose sight of the pivotal role of repeated hands-on practice to ensure proper psychomotor skills. Also note that our framework does not exclusively pertain to NLS; it is probably also useful for the improvement of adherence to pediatric and adult resuscitation guidelines.

Our research group is currently working on follow-up studies, in which one or more strategies, taken from our framework, will be employed in an attempt to improve resuscitation guideline compliance. A very promising strategy, in our opinion, is the application of electronic decision support tools [4, 18]. We are planning to implement such a tool at our department, and will hopefully report on its use during delivery room resuscitation in the near future. A bundle of interventions from our framework will be used in a study that explores ways to enhance adherence to the airway, breathing, circulation, disability, and exposure (ABCDE) algorithm during the assessment of critically ill patients. Finally, we are investigating if use of a mixed reality device (i.e., the Microsoft® HoloLens™) will improve NLS guideline adherence.

### Strengths

The main strength of our study was the use of an adequately calibrated, model-driven, high fidelity neonatal patient simulator. This enabled us to obtain objective, quantitative data on important resuscitation quality metrics, including CC rate and effectiveness. Inasmuch as our participants did not prepare for participation in the NLS scenario and knowledge assessment, our findings reflect the ad hoc NLS capabilities of Dutch general pediatricians. Furthermore, participants worked in 24 different hospitals located in different regions of the Netherlands, so our results are probably representative of the quality of NLS performance in our country.

### Limitations

This study took place prior to the implementation of the 2015 ERC guidelines [8], so we used the 2010 ERC guideline as a reference [1]. However, we believe that the changes in the latest version of the guideline do not affect the interpretation of our results, and that our conclusions still apply today. We used a locally adapted neonatal patient simulator, because this simulator is customized to our needs. Although this may preclude reproducibility, we expect our measurements to be repeatable and replicable, since we appropriately calibrated our simulator. UVC insertion was not tested, though pediatricians were asked to perform this real-time. The MCQ and checklist were developed with care, but not formally validated. The MCQ used to assess NLS knowledge on ERC courses has not been officially validated either. Using a test from abroad (e.g., the NRP knowledge assessment) would have required a separate validation study first. Also, due to methodological differences between the NLS and NRP, the NRP test will probably not be suitable to assess NLS knowledge.

## Conclusions

The adherence of Dutch general pediatricians to the NLS guideline was suboptimal in our simulated setting. Their knowledge of important aspects of resuscitation at birth also requires attention. In an attempt to improve resuscitation guideline adherence, we constructed a comprehensive framework containing multiple suggestions to this end.

## Electronic supplementary material

ESM 1 (PDF 67 kb)

## Abbreviations

ABCDE: Airway, breathing, circulation, disability, exposure
AHA: American Heart Association
APLS: Advanced Pediatric Life Support
CME: Continuing medical education
CPR: Cardiopulmonary resuscitation
EPALS: European Pediatric Advanced Life Support
ERC: European Resuscitation Council
IQR: Interquartile range
MCQ: Multiple-choice questions
NLS: Neonatal Life Support
NRP: Neonatal Resuscitation Program
PIP: Peak inspiratory pressure
UVC: Umbilical venous catheter
