# Supplementary material for: Neonatal resuscitation guideline adherence: simulation study and framework for improvement
Source: Eur J Pediatr. 2020 May 29;179(11):1813–22. doi: 10.1007/s00431-020-03693-6 (PMC7547969; doi:10.1007/s00431-020-03693-6)
Supplement: Supplementary file 1 — (PDF 67 kb) [file 431_2020_3693_MOESM1_ESM.pdf]

Electronic Supplementary Material 1

Article title: Neonatal resuscitation guideline adherence: simulation study and framework for improvement

Journal: European Journal of Pediatrics

Authors: Binkhorst M, van de Wiel I, Draaisma JMT, van Heijst AFJ, Antonius T, Hogeveen M

Correspondence to: Mathijs Binkhorst, Radboud Institute for Health Sciences (RIHS), Department of Neonatology (804), Radboud University Medical Center Amalia Children's Hospital. E-mail: Mathijs.Binkhorst@radboudumc.nl

Knowledge test with results (n=45)

Correct answers are written in bold font.

No.	Question	Correct
1	Below which gestational age is plastic wrapping recommended?<br>a. < 26 weeks<br>b. < 27 weeks<br>**c. < 28 weeks**<br>d. < 29 weeks	84.4%
2	What are the possible consequences of hypothermia directly after birth?<br>a. Increased mortality<br>b. Metabolic acidosis<br>c. Elevated pCO_2<br>**d. Answers a. and b. are correct**	80.0%

3	Below which heart rate is it unreliable to feel cord pulsations?<br><br>a. 120 beats per minute<br><br>**b. 100 beats per minute**<br><br>c. 80 beats per minute<br><br>d. 60 beats per minute	57.8%
4	Which of the following statements is/are correct?<br><br>1. Colour is a reliable method to judge oxygenation.<br><br>2. Colour assessment is essential to proceed in the NLS algorithm.<br><br>a. Statement 1 is correct, statement 2 is incorrect<br><br>b. Statement 2 is correct, statement 1 is incorrect<br><br>c. Both statements are correct<br><br>**d. Both statements are incorrect**	80.0%
5	Which of the following statements is/are correct?<br><br>1. The correct head position of a newborn is the neutral position.<br><br>2. Chin lift and jaw thrust are airway opening maneuvers in a newborn.<br><br>a. Statement 1 is correct, statement 2 is incorrect<br><br>b. Statement 2 is correct, statement 1 is incorrect<br><br>**c. Both statements are correct**<br><br>d. Both statements are incorrect	95.6%
6	How to determine the correct size of an oropharyngeal airway?<br><br>a. Distance from the tip of the nose to the tragus<br><br>**b. Distance from the center of the mouth to the angle of the jaw**<br><br>c. Distance from the corner of the mouth to the angle of the jaw<br><br>d. Distance from the center of the mouth to the tragus	77.8%
7	Above which gestational age/weight can a LMA be considered?<br><br>a. Newborns weighing ≥ 2500 grams<br><br>**b. Newborns weighing ≥ 2000 grams**	33.3%

	c. Newborns with a GA ≥ 37 weeks<br><br>d. Newborns with a GA ≥ 32 weeks	
8	How should the initial inflation breaths be performed?<br><br>a. Five inflations during 3 seconds each with a PIP of 30 cm H_2O<br><br>b. Five inflations during 5 seconds each with a PIP of 30 cm H_2O<br><br>**c. Five inflations during 3 seconds each with a PIP of 20 cm H_2O**<br><br>d. Five inflations during 5 seconds each with a PIP of 20 cm H_2O	82.2%
9	What is the correct rate of ventilations in the absence of spontaneous breathing?<br><br>**a. 30-60 breaths per minute**<br><br>b. 20-40 breaths per minute<br><br>c. 40-60 breaths per minute<br><br>d. 20-60 breaths per minute	35.6%
10	What is an acceptable pre-ductal oxygen saturation at 5 min?<br><br>a. 75%<br><br>b. 80%<br><br>**c. 85%**<br><br>d. 90%	66.7%
11	Which of the following statements is/are correct?<br><br>1. The pulse oximeter sensor should be placed on the right hand/wrist.<br><br>2. Connect the sensor to the pulse oximeter before placing the sensor on the right hand/wrist.<br><br>**a. Statement 1 is correct, statement 2 is incorrect**<br><br>b. Statement 2 is correct, statement 1 is incorrect<br><br>c. Both statements are correct<br><br>d. Both statements are incorrect	66.7%
12	Which of the following statements is/are correct?<br><br>1. The correct compression:ventilation ratio is 3:1.	40.0%

	2. One should aim for approximately 100 events<br>(i.e. compressions plus ventilations) per minute.<br>**a. Statement 1 is correct, statement 2 is incorrect**<br>b. Statement 2 is correct, statement 1 is incorrect<br>c. Both statements are correct<br>d. Both statements are incorrect	
13	Below which heart rate should chest compressions be started?<br>a. < 100 beats per minute<br>b. < 80 beats per minute<br>**c. < 60 beats per minute**<br>d. < 40 beats per minute	82.2%
14	When should the FiO_2 be increased, if not already done before? [a]<br>a. At the start of assisted ventilation<br>**b. At the start of chest compressions**<br>c. If the oxygen saturation is < 85% after 5 minutes<br>d. In the absence of chest rise	75.6%
15	What is the correct dose of epinephrine?<br>a. 10 micrograms per kilogram i.v.<br>b. 50-100 micrograms per kilogram e.t.<br>c. 10 micrograms per kilogram i.o.<br>**d. All of the above**	68.9%
16	What is the recommended administration route of epinephrine?<br>a. Endotracheal<br>b. Intraosseous<br>c. Intravenous, through a peripheral cannula<br>**d. Intravenous, through an umbilical venous catheter**	95.6%

17	In which babies is delayed cord clamping (1 min) recommended?<br><br>a. Uncompromised preterm infants<br><br>b. Uncompromised term infants<br><br>**c. Answers a. and b. are correct**<br><br>d. Term and preterm infants, irrespective of their condition	40.0%

Legend

FiO_2, fraction of inspired oxygen; GA, gestational age; LMA, laryngeal mask airway; NLS, newborn life support; pCO_2, partial pressure of carbon dioxide; PIP, peak inspiratory pressure.

[a] Although not evidence-based, the administration of supplementary oxygen at the start of chest compressions is considered to be 'sensible' according to the ERC guideline and it is an actual prescription in the Dutch NLS guideline.
